# Genome-Wide Analysis of AGPase Identifies *CsAGP4* as a Regulator of Watermelon Mosaic Virus Resistance in Cucumber

**DOI:** 10.3390/ijms27114764

**Published:** 2026-05-25

**Authors:** Xiao Sun, Jiantao Guan, Han Miao, Xiaoping Liu, Xingfang Gu, Shaoyun Dong, Shengping Zhang

**Affiliations:** 1 State Key Laboratory of Vegetable Biobreeding, Institute of Vegetables and Flowers, Chinese Academy of Agricultural Sciences, Beijing 100081, China; 2 National Nanfan Research Institute (Sanya), Chinese Academy of Agricultural Sciences, Sanya 572024, China

**Keywords:** cucumber, AGPase, genome, antiviral defense, watermelon mosaic virus

## Abstract

The ADP-glucose pyrophosphorylase (AGPase) gene family plays an essential role in starch metabolism and stress adaptation. However, its function in antiviral defense remains largely uncharacterized. Cucumber (*Cucumis sativus* L.), a globally important vegetable crop, frequently experiences severe yield losses due to viral infections. In this study, we systematically identified five AGPase genes in cucumber, categorizing them into large and small subunits. Analysis of conserved motifs revealed ten conserved sequences, with the NTP (Nucleoside Triphosphate) transferase domain representing a signature feature of the *AGPase* family. Promoter regions contained multiple cis-regulatory elements associated with stress responses and hormone signaling. Transcriptomic profiling revealed tissue-specific expression patterns of *CsAGP* genes, with pronounced enrichment in leaves. Notably, *CsAGP2*, *CsAGP4*, and *CsAGP5* were strongly induced under biotic and abiotic stresses. Of these, *CsAGP4* exhibited rapid, transient induction specifically in the virus-resistant line ‘228’, but not in the susceptible line ‘65G’. Hormonal treatments showed that abscisic acid (ABA) rapidly activated most *CsAGP* genes and acted synergistically with viral infection to amplify *CsAGP4* expression. Functional analysis via CRISPR/Cas9-mediated knockout of *CsAGP4* revealed that the mutation disrupted starch granule formation and significantly altered resistance to watermelon mosaic virus (WMV) in ‘Poinsett 97’. Our work provides a systematic characterization of the AGPase gene family in cucumber and establishes its role in defense responses. Importantly, we identify *CsAGP4* as a positive regulator of antiviral immunity, highlighting its potential as a target for breeding virus-resistant cucumber varieties.

## 1. Introduction

Cucumber (*Cucumis sativus* L.) is cultivated extensively across the world and represents a major vegetable commodity in both economic and agricultural terms [1,2], accounting for 59.5% of the global cultivation area and 80.8% of the total yield (http://faostat.fao.org, accessed on 10 April 2025). However, during production, cucumber plants face escalating challenges from viral pathogens, including watermelon mosaic virus (WMV), cucumber mosaic virus (CMV), and zucchini yellow mosaic virus (ZYMV). Infection by these viruses commonly causes chlorosis, mosaic symptoms, and stunted growth, leading to substantial yield losses [3,4].

Starch is a primary carbon storage polysaccharide in plants that plays a dual role in energy homeostasis and stress adaptation. Its synthesis depends on the coordinated action of several enzymes, including ADP-glucose pyrophosphorylase (AGPase), granule-bound starch synthase (GBSS), soluble starch synthase (SS), starch branching enzyme (SBE), and debranching enzyme (DBE) [5]. Among them, AGPase functions as the key rate-limiting enzyme because it converts glucose-1-phosphate and ATP into ADP-glucose, thereby exerting a strong influence on the rate of starch production and final starch accumulation.

Plant ADP-glucose pyrophosphorylase (ADP-Glc PPase) is a heterotetrameric enzyme consisting of two large subunits (APL) and two small subunits (APS), and each subunit contains the characteristic NTP-transferase (PF00483) domain. Enzymatic activity is stimulated by 3-phosphoglycerate and suppressed by inorganic phosphate, whereas additional metabolites such as fructose-6-phosphate may further influence activity in certain species or tissues [6,7]. The small (APS) and large (APL) subunits of ADP-Glc PPase are homologous proteins. In plants, the small subunit is typically encoded by one or two genes, whereas the large subunit is often represented by multiple paralogs that show tissue- or plastid-specific expression [8]. Therefore, compared to small subunits, the specific spatio-temporal expression pattern of AGPase genes is mainly determined by large subunits [9,10]. The AGPase family has already been described in a range of monocot and dicot species, including *Arabidopsis thaliana* (four APLs and two APSs), *Zea mays* (four APLs and two APSs), *Oryza sativa* (four APLs and three APSs), *Solanum lycopersicum* (three APLs and one APS), *Manihot esculenta Crantz* (four APLs and three APSs), and *Musa acuminata* L. (six APLs and two APSs) [11].

Beyond their well-known functions in plant growth and development, many *AGPase* genes are responsive to diverse biotic and abiotic stresses, including heat, cold, salinity, drought, and infection by phytopathogens [12,13,14]. During the maturation stage, heat stress on the AGPase enzyme seriously affects the biosynthesis of starch in rice [15]. In downy mildew-infected grapevine leaves, increased AGPase activity leads to starch accumulation [16]. Moreover, in melon (*Cucumis melo*), CMV infection downregulates the expression level of AGPase, which leads to decreased starch content and compromised antiviral defense [17]. However, whether and how AGPase responds to viral infection in cucumber remains largely unknown.

The plant defense system is coordinately regulated by the interplay between sugar metabolism and phytohormone signaling pathways [18,19]. Increasing evidence suggests that AGPase-dependent starch metabolism is embedded in hormone-mediated immune regulation, indicating that AGPase may connect metabolic adjustment with defense activation. In *Arabidopsis*, ABA not only induces AGPase expression under drought stress to stabilize carbon reserves [20] but also enhances starch accumulation by phosphorylating AGPase subunits via SnRK2 kinases, thereby optimizing energy allocation for survival [8,21]. Exogenous jasmonic acid (JA) treatment greatly activates the defense of *Rosa chinensis* against *Botrytis cinerea* [22]. Exogenous addition of methyl jasmonate (MeJA) to citrus fruits significantly enhances the activities of PEROX and polyphenol oxidase (PPO), thereby effectively inhibiting the occurrence of *Penicillium digitatum* and *Alternaria alternata* [23]. In addition, silencing of MeJA- and methyl salicylate (MeSA)-related genes led to significantly increased susceptibility to tobacco mosaic virus (TMV) in *Nicotiana benthamiana* [24].

Although extensive studies have revealed the importance of AGPase in plant stress responses, the functional mechanism of the *CsAGP* gene family in antiviral defense is still unknown. Here, we performed a comprehensive study of the *AGPase* family in cucumber, defining its membership, structure, and regulatory landscapes. Through comprehensive analyses involving transcriptomics, hormone application, and pathogen infection, we observed that *CsAGP4* undergoes dynamic regulation. CRISPR/Cas9-mediated knockout of *CsAGP4* confirmed its necessity for starch granule formation and virus resistance. These results indicate that *CsAGP4*-mediated starch metabolism is a positive facet of the plant antiviral response.

## 2. Results

### 2.1. Identification of the AGPase Gene Family in Cucumber

Using a combination of HMM and BlastP-based tools, we identified five *AGPase* genes in the cucumber genome (Table 1). Predicted subcellular localization placed all five proteins in the chloroplast. To further understand the structural and functional attributes of the *CsAGP* gene family in cucumber, we conducted a comprehensive analysis of their encoded proteins. The encoded proteins ranged from 521 to 536 amino acids in length, with a mean length of 527.8 amino acids. Their predicted molecular masses spanned 56,917.85 to 59,906.89 Da, and their theoretical isoelectric points ranged from 5.86 to 9.09. Overall, these parameters indicate that members of the *CsAGP* family share broadly conserved biochemical characteristics.

### 2.2. Phylogenetic and Structural Analysis of AGPase Proteins in Cucumber

**Figure 1:**
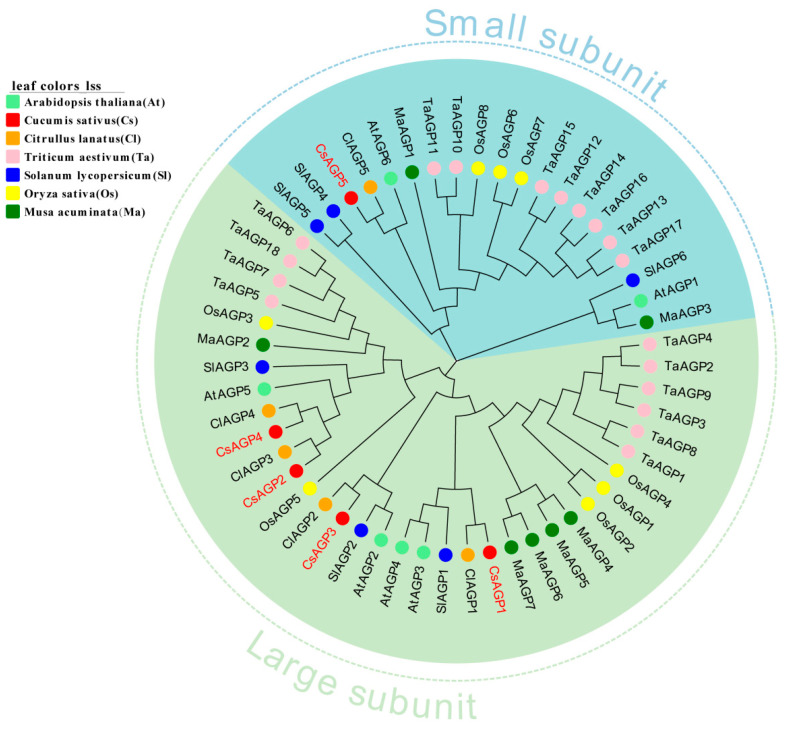
Phylogenetic analysis of *AGPases* from cucumber, *Arabidopsis*, rice, watermelon, tomato, wheat, and banana. The neighbor-joining tree was constructed using MEGA X with 1000 bootstraps. Two subgroups were identified and classified according to large-subunit and small-subunit type. The red, light green, orange, light blue, pink, yellow and green represent AGPase proteins from cucumber, *Arabidopsis*, watermelon, tomato, wheat, rice, and banana, respectively.

**Figure 2:**
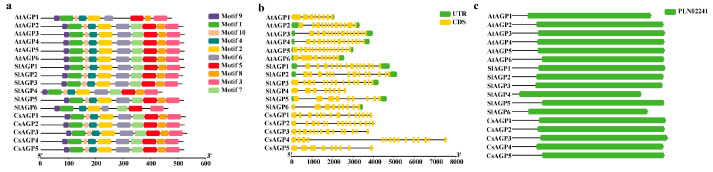
Analysis of conserved motifs and gene structure of *AGPases*. (**a**) Conserved motifs in different species (*Arabidopsis*, tomato, cucumber). The motifs, numbered 1–10, are indicated in different colors. (**b**) The yellow boxes indicate exons. Black lines represent introns. Green boxes represent the untranslated regions (UTRs). (**c**) NTP transferase (PLN02241) domains of AGPase in different species.

To clarify the evolutionary relationships of cucumber AGPases, we constructed a phylogenetic tree based on five *CsAGP* (*Cucumis sativus* L.), six *AtAGP* (*Arabidopsis thaliana*), seven *MaAGP* (*Musa acuminata* L.), eight *OsAGP* (*Oryza sativa* L.), nine *SlAGP* (*Solanum lycopersicum* L.), eighteen *TaAGP* (*Triticum aestivum* L.) and five *ClAGP* (*Citrullus lanatus* L.) proteins. As shown in Figure 1, the proteins separated into two major branches corresponding to the large and small subunits; group II contained 35 large-subunit members, whereas group I comprised 20 small-subunit members. It is clear that the five *AGPase* genes in cucumber are closely related to the five *AGPase* genes in watermelon, and four of the five *CsAGP genes* belong to the large subunit. In comparison, their phylogenetic relationships with *Arabidopsis thaliana* and tomato *AGPase* genes are relatively more distant. In order to further investigate the structural features and evolutionary mechanisms of *AGPase* gene families, the conserved protein motifs (Figure 2a) and gene structures (Figure 2b) were comparatively analyzed in the genomes of cucumber, *Arabidopsis*, and tomato. MEME analysis identified 10 motifs that were retained in most AGPase proteins. Most of the conserved motifs were evenly distributed across genes in three different species. The distribution of conserved motifs diverged significantly among the different species. Furthermore, the *AGPase* gene family was identified by the presence of an NTP transferase domain (PLN02241, Figure 2c), which is characteristic of the AGPase protein family. These findings provide additional evidence for specific evolutionary characteristics of *AGPase* genes across plant species.

### 2.3. Genomic Organization and Evolutionary Analysis of CsAGP Genes

**Figure 3:**
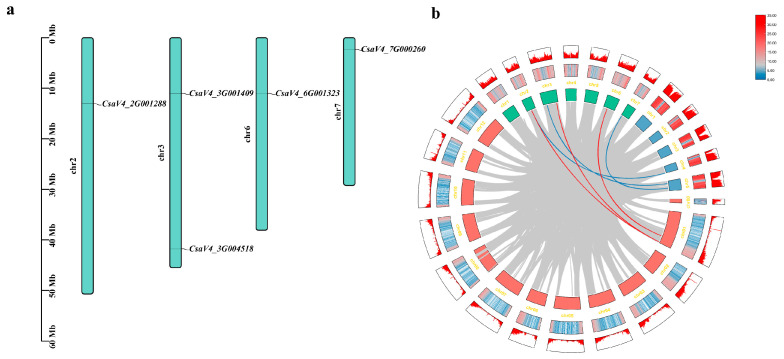
Chromosomal distribution and collinearity analysis of *CsAGP genes*. (**a**) Chromosomal distribution of *CsAGP* genes in cucumber. Light green lines represent different chromosomes, and marks on them indicate the corresponding genes and their locations. (**b**) Collinearity analysis of *CsAGP genes* in cucumber. Homologous gene pairs in *Arabidopsis* (blue), cucumber (green), and tomato (red). The red and blue lines represent the homologous pairs of the AGPase gene family.

As illustrated in Figure 3a, five *CsAGP* genes were distributed across four chromosomes. There was one *CsAGP gene* on chromosome 2, one on chromosome 6, and one on chromosome 7, while two *CsAGP genes* were located on chromosome 3. The two *CsAGP genes* on chromosome 3 may indicate a gene duplication event or a potential hotspot for genes involved in starch biosynthesis and stress responses, which could provide insight into the functional diversity of the *CsAGP* family. Because gene duplication often promotes adaptation to changing environments, including responses to biotic challenges, this arrangement may be biologically meaningful. Collinearity analysis of the *AGPase* gene family in cucumber identified three pairs between cucumber and tomato and two pairs between cucumber and *Arabidopsis* (Figure 3b). This pattern suggests that these *CsAGP* genes may have been evolutionarily conserved across species.

### 2.4. Cis-Regulatory Element Profiling of CsAGP Gene Promoter

**Figure 4:**
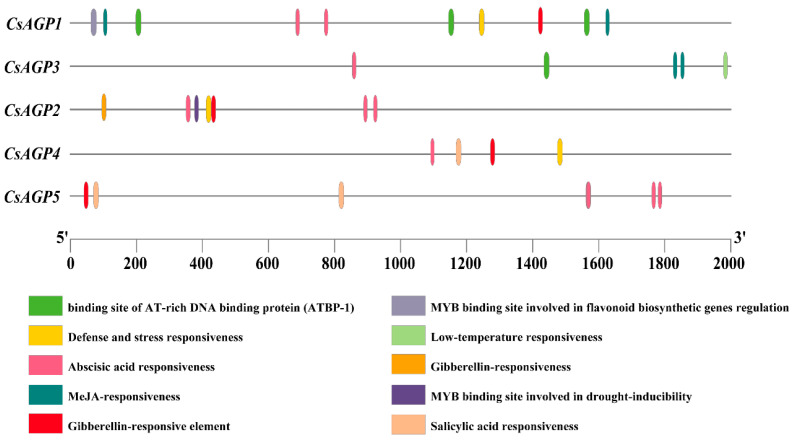
Putative cis-acting regulatory elements (CAREs) of the *AGPase* gene family. The CARE analysis was conducted on the 2 kb upstream region using the PlantCARE online server. The green CARE represents the binding site of the AT-rich DNA-binding protein (ATBP-1). The yellow CARE represents a cis-acting element involved in defense and stress responsiveness. The pink CARE represents a cis-acting element involved in abscisic acid responsiveness. The dark green CARE represents a cis-acting regulatory element involved in MeJA responsiveness. The red CARE represents a gibberellin-responsive element. The gray CARE represents an MYB binding site involved in the regulation of flavonoid biosynthetic genes. The light green CARE represents a cis-acting element involved in low-temperature responsiveness. The orange CARE represents a cis-acting element involved in gibberellin responsiveness.

To further investigate the regulatory mechanisms of *CsAGP* genes and their modulation by phytohormones, defense signaling, and stress responses, we analyzed the 2000 base pair upstream promoter regions of *CsAGP* genes to identify cis-acting regulatory elements (CAREs). Numerous CAREs were detected, including motifs associated with light responsiveness, MeJA signaling, ABA responsiveness, salicylic acid signaling, and general defense or stress responses. Among these, light-, MeJA-, and ABA-related elements were the most abundant, underscoring their potential role in regulating *CsAGP* function in plant growth and development. Additionally, we identified numerous stress-responsive elements (Figure 4), including those responsive to low temperature and drought. This suggests that *CsAGP* genes not only regulate essential metabolic pathways but also contribute to enhancing plant resilience to both biotic and abiotic stresses.

Promoter analysis reveals that *CsAGP* genes are orchestrated by a network of phytohormones and stress-related signaling pathways. Together, these results indicate that *CsAGP* genes function as key regulators in coordinating plant responses to environmental challenges, a process critical for maintaining metabolic balance and improving overall plant fitness.

### 2.5. Expression Profiling of CsAGP Genes During Development and Under Diverse Stress Conditions

**Figure 5:**
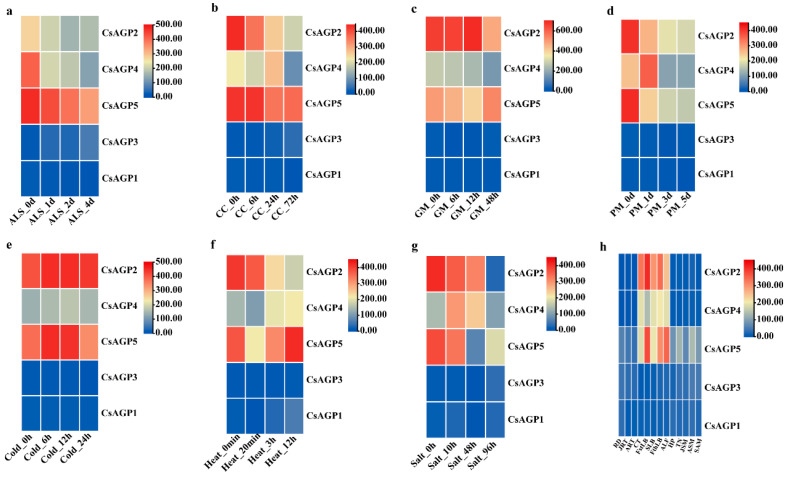
Expression profiling of *CsAGP* genes during development and under diverse stress conditions. (**a**) The heatmap of cucumber *CsAGP* gene family expression at 0, 1, 2, and 4 days post-inoculation with bacterial angular leaf spot. (**b**) The heatmap of cucumber *CsAGP* gene family expression at 0, 6, 24, and 72 h post-inoculation with Cladosporium cucumerinum. (**c**) The heatmap of cucumber *CsAGP* gene family expression at 0, 6, 12, and 48 h post-inoculation with gray mold. (**d**) The heatmap of cucumber *CsAGP* gene family expression at 0, 1, 3, and 5 days post-inoculation with powdery mildew. (**e**) The heatmap of cucumber *CsAGP* gene family expression at 0, 6, 12, and 24 h under cold stress. (**f**) The heatmap of cucumber *CsAGP* gene family expression at 0 h, 20 min, 3 h, and 12 h under heat stress. (**g**) The heatmap of cucumber *CsAGP* gene family expression at 0, 10, 48, and 96 h under salt stress. (**h**) The heatmap of cucumber *CsAGP* gene family expression in different tissues, including radicle, juvenile root, adult root, cotyledon, first leaf blade, second leaf blade, fourth leaf blade, adult leaf, hypocotyl, tendril, juvenile stem, adult stem, and shoot apical meristem.

To explore the relationship between *CsAGP* genes and biotic/abiotic stress responses, we analyzed expression changes in the cucumber variety ‘9930’ under multiple stress conditions, including Cladosporium cucumerium (CC), gray mold (GM), powdery mildew (PM), angular leaf spot (ALS), salt stress, cold stress, and heat stress (Figure 5a–g). Based on heatmap analysis of transcriptome data, we found that *CsAGP2*, *CsAGP4*, and *CsAGP5* were significantly induced when the plants experienced biotic and abiotic stresses, showing notable changes in expression levels, whereas *CsAGP1* and *CsAGP3* showed no significant changes. These analyses revealed dynamic *CsAGP* expression patterns in response to pathogenic infections and environmental stressors, highlighting their potential roles in plant defense and metabolic adaptation.

Furthermore, we characterized expression patterns across cucumber developmental stages and tissues. *CsAGP* genes were expressed predominantly in leaves, with particularly strong transcript accumulation for *CsAGP2*, *CsAGP4* and *CsAGP5* (Figure 5h). By mapping these expression changes across different growth stages, we elucidated their functional significance in starch biosynthesis, disease resistance, and stress adaptation mechanisms.

### 2.6. Expression Profiling of CsAGP Genes in Resistant and Susceptible Cucumbers in Response to Pathogen Infection

To compare the behavior of *CsAGP* genes in resistant and susceptible backgrounds under pathogen stress, we analyzed their expression levels in leaves following infection by several severe pathogens, including gummy stem blight (GSB), angular leaf spot (ALS), and watermelon mosaic virus (WMV).

**Figure 6:**
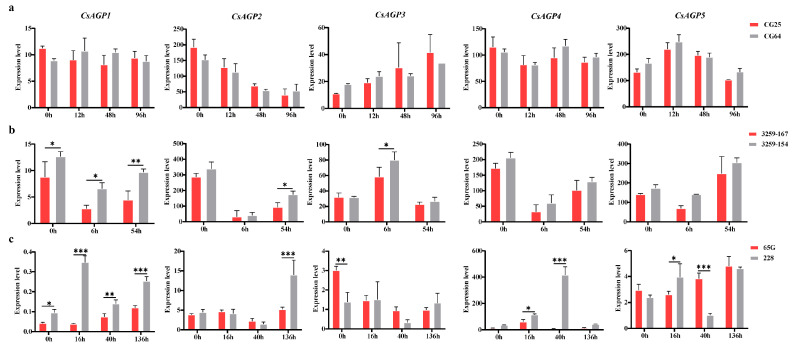
The expression levels of *CsAGP* genes under different disease stresses. (**a**) Expression analysis of *CsAGP* genes in ‘CG64’ and ‘CG25’ under GSB infection. (**b**) Expression analysis of *CsAGP* genes in ‘3259–154’ and ‘3259–167’ under ALS infection. (**c**) Expression analysis of *CsAGP* genes in ‘228’ and ‘65G’ under WMV infection. A one-way analysis of variance (ANOVA) was conducted, followed by Tukey’s HSD test. Data represent mean ± SD of three biological replicates. *, ** and *** represent data that differ significantly using a two-tailed Student’s *t*-test at *p* < 0.05, *p* < 0.01 and *p* < 0.001, respectively.

For GSB, the susceptible line ‘CG25’ developed obvious lesions at 3 to 5 days post-inoculation (dpi), whereas the resistant line ‘CG64’ displayed no obvious symptoms. Upon GSB infection, *CsAGP2* expression gradually decreased in both resistant and susceptible lines, while *CsAGP3* expression increased progressively. *CsAGP5* showed increased expression at 0 h and 12 h post-inoculation (hpi), and then gradually declined (Figure 6a).

For ALS infection, the susceptible line ‘3259–167’ exhibited symptoms at 1 dpi. All four analyzed *CsAGP* genes displayed similar expression patterns in both susceptible and resistant materials. Notably, *CsAGP3* expression in both lines increased continuously from 0 h to 6 hpi before gradually returning to basal levels, a pattern potentially associated with the observed dryness symptoms (Figure 6b).

Following WMV inoculation, the resistant line ‘228’ showed continuous and significant upregulation of *CsAGP1* and *CsAGP5* expression. *CsAGP4* expression increased from 0 to 40 hpi and then decreased. In contrast, the susceptible line ‘65G’ exhibited no significant change in *CsAGP4* expression. These findings suggest that *CsAGP4* may play a positive role in antiviral mechanisms (Figure 6c).

### 2.7. Modulation of CsAGP Expression by ABA and JA Under Viral Infection

Given that abscisic acid (ABA) and jasmonic acid (JA) are well-established key regulators of plant antiviral responses—mediating defense signaling and enhancing resistance—we investigated whether exogenous applications of these hormones could modulate the expression of the *CsAGP* gene family. Specifically, we designed experiments to assess changes in *CsAGP* gene expression patterns following hormone treatment, aiming to elucidate potential functional linkages between these phytohormones and the *CsAGP* genes within plant antiviral mechanisms.

**Figure 7:**
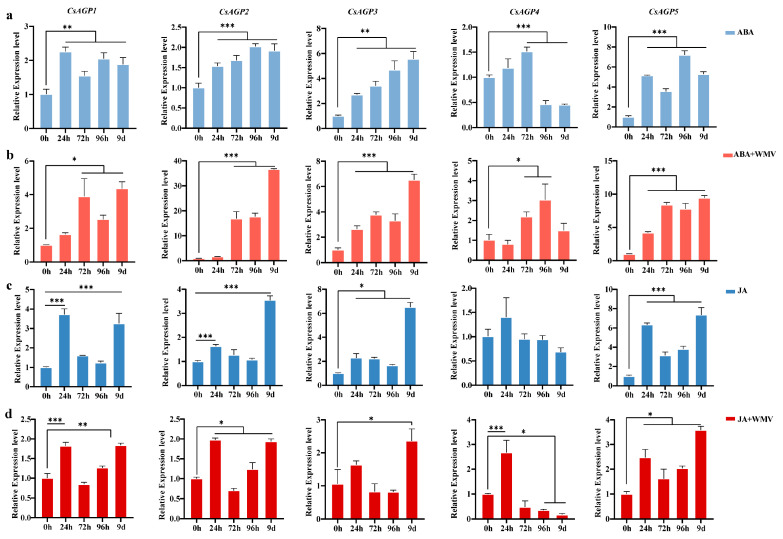
Expression pattern of five *CsAGP* genes in response to abscisic acid (ABA) or jasmonic acid (JA) treatment of WMV-infected cucumber seedlings. (**a**) Analysis of the changes in expression levels of *CsAGP* genes upon ABA application in the absence of WMV stress. (**b**) Analysis of the changes in expression levels of *CsAGP* genes under WMV stress with concurrent ABA treatment. (**c**) Analysis of the changes in expression levels of *CsAGP* genes upon JA application in the absence of WMV stress. (**d**) Analysis of the changes in expression levels of *CsAGP* genes under WMV stress with concurrent JA treatment. The response is indicated by vertical bars across five time points for ABA (0.1 mM), WMV + ABA (0.1 mM), JA (0.1 mM) and WMV + JA (0.1 mM) treated samples and is labeled on the horizontal axis. A one-way analysis of variance (ANOVA) was conducted, followed by Tukey’s HSD test. Data represent mean ± SD of three biological replicates. *, ** and *** represent data that differ significantly using a two-tailed Student’s *t*-test at *p* < 0.05, *p* < 0.01 and *p* < 0.001, respectively.

The expression levels of *CsAGP* genes exhibited dynamic changes in response to ABA and JA treatments. Exogenous spraying experiments were conducted on leaves of the cucumber line ‘228’, both with and without WMV inoculation. The results are summarized as follows. Group I (ABA Treatment, Figure 7a): Compared to the untreated control, *CsAGP1*, *CsAGP2*, *CsAGP3*, and *CsAGP5* genes showed significantly upregulated expression upon ABA treatment. In contrast, the expression of *CsAGP4* increased gradually from 0 to 72 hpi before rapidly declining to levels below those of the control. Group II (ABA and WMV Treatment, Figure 7b): Following WMV inoculation, exogenous ABA application resulted in a consistent expression pattern for *CsAGP1*, *CsAGP2*, *CsAGP4*, and *CsAGP5*, with expression levels at each time point being higher than those in Group I. Notably, the expression of *CsAGP3* remained unchanged. This suggests that ABA induces the upregulation of this gene family in response to WMV infection, further indicating a potential interaction between viral infection and the ABA signaling pathway. Group III (JA Treatment, Figure 7c): The regulatory pattern of *CsAGP* genes was largely consistent with that observed in the ABA treatment group, differing primarily in response timing. This suggests that the gene family exhibits a similar response mechanism to both hormones. Group IV (JA and WMV Treatment, Figure 7d): Similar to the pattern in Group II, gene expression was altered under JA treatment in the presence of viral infection. While the overall expression trend for all genes was similar to that in Group III, *CsAGP4* showed a slightly higher expression level at 24 hpi, whereas the overall expression levels of the other four genes were significantly lower than in Group III. This marked the most distinct difference compared to Group II. The differential expression patterns of *CsAGP* genes under ABA and JA treatments highlight the intricate regulatory mechanisms plants employ to cope with stress. These findings provide valuable insights into how ABA and JA regulate *CsAGP* gene expression and starch metabolism in cucumber, as well as their roles in stress responses.

### 2.8. Functional Validation of CsAGP4 in Starch Biosynthesis and Antiviral Defense

**Figure 8:**
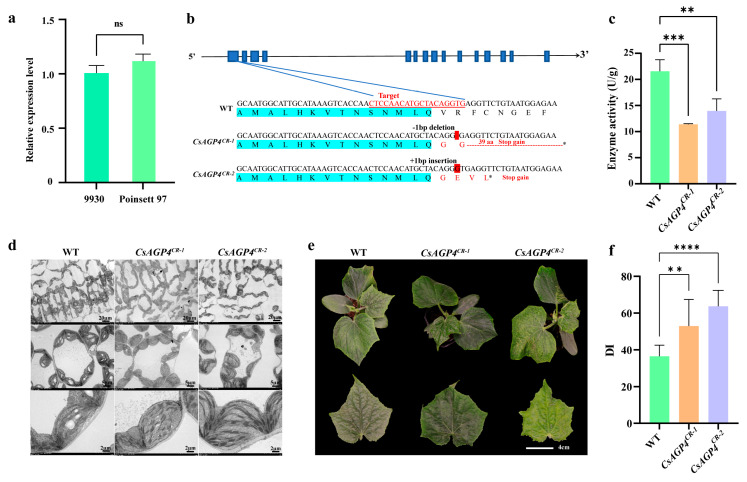
Functional validation of the *CsAGP4* gene. (**a**) *CsAGP4* expression analysis in ‘Poinsett 97’ and ‘9930’. Not significant (ns, *p* ≥ 0.05). (**b**) CRISPR/Cas9 technology was used to edit the target sequence of the *CsAGP4* gene and the sequence of two homozygous mutants. (**c**) ADPGase enzyme activity in wild type and knockout lines (*CsAGP4^CR-1^* and *CsAGP4^CR-2^*) was measured at the seedling stage using an enzyme activity assay kit. (**d**) Transmission electron microscopy (TEM) analysis of starch granule accumulation in wild type and two *CsAGP4* knockout lines. Scale bar: 20 μm, 5 μm, and 2 μm, respectively. (**e**) Observations of WMV symptoms in wild type and two independent *CsAGP4* knockout lines (*CsAGP4^CR-1^* and *CsAGP4^CR-2^*) at 9 days post−inoculation (dpi). Scale bar: 4 cm. (**f**) Wild−type and mutant plants were inoculated, and the disease index was assessed at 9 days post−inoculation (dpi). A one−way analysis of variance (ANOVA) was conducted, followed by Tukey’s HSD test. Data represent mean ± SD of three biological replicates. *, **, *** and **** represent data that differ significantly using a two−tailed Student’s *t*−test at *p* < 0.05, *p* < 0.01 and *p* < 0.001, *p* < 0.0001, respectively.

We selected the ‘Poinsett 97’ accession for subsequent gene editing. Sequence analysis showed that the coding sequence of *CsAGP4* in this accession is identical to that of the reference genome ‘9930’ (Figure S1). Moreover, *CsAGP4* exhibited similar transcriptional levels to those in ‘9930’ (Figure 8a) and retained AGPase activity in this accession (Figure 8c), confirming that *CsAGP4* is functionally competent in ‘Poinsett 97’. Using this accession as the background, we generated two homozygous mutant lines (*CsAGP4^CR-1^* and *CsAGP4^CR-2^*) via CRISPR/Cas9-mediated genome editing (Figure 8b).

Since *CsAGP4* encodes a key rate-limiting enzyme in starch biosynthesis, we next examined whether its disruption affects starch accumulation. Transmission electron microscopy (TEM) revealed that starch granules were nearly absent in the leaves of both mutants compared with the wild type (Figure 8d). Consistently, AGPase activity was significantly reduced in the mutants (Figure 8c), confirming that *CsAGP4* mutation impairs starch biosynthesis. We next assessed the susceptibility of wild-type and mutant plants to WMV infection. At 9 dpi, both mutant lines developed more severe disease symptoms than the wild type, with disease indices of 60 and 58 in *CsAGP4^CR-1^* and *CsAGP4^CR-2^*, respectively, both significantly higher than those of the wild type (Figure 8e,f). Collectively, these results demonstrate that *CsAGP4* plays a positive role in plant resistance against WMV.

## 3. Discussion

In this work, we performed a systematic analysis of the *AGPase* gene family in cucumber and identified five *CsAGP* members distributed across four chromosomes. Phylogenetic clustering divided *CsAGP genes* into two clades corresponding to large and small subunits, consistent with previous classifications in *Arabidopsis*, rice, and banana [5,11]. Notably, *CsAGP genes* showed closer evolutionary relationships with watermelon homologs. The uneven chromosomal distribution of *CsAGP* genes, especially the tandem duplication on chromosome 3, points to lineage-specific expansion that may have contributed to functional redundancy and diversification. Such duplications are common in stress-responsive gene families and enable plants to adapt to environmental challenges [14]. The conserved NTP transferase domains and motif architectures across species further underscore the indispensable role of AGPase in starch biosynthesis. However, the divergence in exon-intron structures and cis-regulatory elements implies species-specific regulatory mechanisms, potentially fine-tuning AGPase activity in response to cucumber-specific stressors like WMV. This lays a foundation for further functional studies of *CsAGP* in cucumber.

The preferential expression of *CsAGP* genes in photosynthetically active tissues (leaves) (Figure 5h) underscores their role in balancing growth and defense. High AGPase activity in leaves ensures rapid starch synthesis during the day, while elevated expression in flowers may support nectar production or pollen development, as seen in maize [15]. The low expression in roots contrasts with findings in cassava, where root-specific AGPases dominate starch storage [25], suggesting tissue-specific subfunctionalization within the family. This divergence may reflect evolutionary adaptations to different sink–source relationships in annual (cucumber) versus perennial (cassava) species.

The interplay between ABA and JA signaling pathways and *CsAGP* expression provides critical insights into hormonal regulation of starch metabolism during viral infection. ABA treatment markedly induced the expression of *CsAGP1*, *CsAGP2*, *CsAGP3*, and *CsAGP5* (Figure 7), in line with reports that ABA promotes AGPase activity in *Arabidopsis* to preserve starch reserves under drought stress [8]. Notably, *CsAGP4* expression (Group II) was synergistically induced by ABA and WMV infection, suggesting a potential link between stress hormone signaling and metabolism. This aligns with emerging evidence that ABA not only regulates stomatal immunity but also reprograms carbohydrate metabolism to limit pathogen resource acquisition [26,27]. Similarly, the JA responsiveness of *CsAGP* may be linked to glycosylation-mediated defense processes, with the *CsAGP* promoter potentially participating in the regulation of defense metabolite accumulation [28,29].

One of the important findings of this study is the differential expression of *CsAGP* genes between virus-resistant (‘228’) and susceptible (‘65G’) genotypes following WMV inoculation. The significant upregulation of *CsAGP4* in resistant plants (Figure 6) aligns with recent reports linking AGPase activity to pathogen defense. For instance, in grapevine, AGPase activity increases during downy mildew infection to bolster starch accumulation, which may restrict pathogen spread by altering carbon partitioning [16]. Similarly, wheat AGPase isoforms are modulated during fungal attacks to redirect resources toward defense metabolites [14]. The combined evidence from enzymatic assays, transmission electron microscopy (TEM), and pathogen inoculation demonstrates that the complete knockout of *CsAGP4* leads to compromised AGPase activity, a reduction in starch granule accumulation, and a significant increase in disease susceptibility (Figure 8). These findings are also consistent with previous studies in grapevine [16] and tomato [30], where starch metabolism was found to enhance plant resistance to pathogens. These results suggest that *CsAGP4* induction in cucumber may promote starch synthesis, which could restrict viral replication by reducing sugar availability or potentially generating soluble glucans that could function as immune-priming signals [31]. Regarding the potential mechanisms by which *CsAGP4*-mediated starch metabolism contributes to antiviral defense, two hypotheses are proposed. On the one hand, starch biosynthesis may act as a carbon sequestration mechanism, restricting the availability of sugars that viruses require for replication. Similar studies have shown that viruses hijack host sugar transporters to sustain their proliferation [32]. On the other hand, because most pathogens draw hexoses, primarily glucose, from the host cytoplasm [33], starch degradation may support host energy metabolism by supplying these sugars, thereby strengthening immune responses to viral invasion [34]. The high starch reserves ensure an adequate energy supply, strengthening the plant’s immunity and overall resistance to viral infection.

Despite providing strong evidence for the role of *CsAGP4*-mediated starch metabolism in antiviral defense, several key questions remain to be explored. First, it is necessary to determine whether *CsAGP4* also confers resistance to other viruses, such as Cucumber mosaic virus (CMV) and Zucchini yellow mosaic virus (ZYMV), to evaluate whether it plays a broader role in plant disease resistance. Furthermore, future studies should investigate whether *CsAGP4* regulates the metabolism of starch to influence viral replication, providing deeper insights into how carbon metabolism modulates virus–host interactions. Furthermore, metabolomic and transcriptomic analyses of wild-type and *CsAGP4* mutant plants during viral infection will be performed to identify key disease-resistance pathways and interacting genes. Additionally, molecular and biochemical assays will be conducted to validate these key interactions, thereby deepening our understanding of plant–virus interaction mechanisms.

## 4. Materials and Methods

### 4.1. Plant Materials and Experimental Design

Eight cucumber inbred lines were used in this study: ‘CG64’ (resistant to Gummy Stem Blight), ‘CG25’ (susceptible to Gummy Stem Blight), ‘3259–154’ (highly resistant to Angular Leaf Spot), ‘3259–167’ (highly susceptible to Angular Leaf Spot), ‘9930’ (a North China-type line), ‘65G’ (sensitive to WMV), ‘228’ (resistant to WMV) and ‘Poinsett 97’ (picked type). All materials were obtained from the Cucumber Research Group, Institute of Vegetables and Flowers, Chinese Academy of Agricultural Sciences (CAAS), Beijing, China, and grown under controlled chamber conditions at Nankou, Beijing. For pathogen inoculation, seedlings were challenged with fungal (GSB), bacterial (ALS), or viral (WMV) pathogens.

Plants were grown under the same controlled chamber conditions, and seedlings were randomly assigned to different treatment groups. For pathogen inoculation and hormone treatment experiments, 21 plants were used per genotype with three biological replicates. At each sampling time point, fully expanded leaves from three plants were pooled as one biological replicate. For qRT–PCR analysis, three biological replicates were used for each time point, and each biological replicate was analyzed with three technical replicates. For AGPase activity assays, three biological replicates were collected for each genotype or treatment, with three plants included in each biological replicate.

### 4.2. Hormone Treatment and Pathogen Inoculation

For hormone treatments, fully expanded first leaves of ‘228’ seedlings were sprayed with 0.1 mM abscisic acid (ABA) or 0.1 mM jasmonic acid (JA) (Solarbio, Beijing, China). Four treatment groups were established: (I) ABA alone, (II) ABA after WMV inoculation, (III) JA alone, and (IV) JA after WMV inoculation. Inoculation of WMV was conducted according to a previous study [35]. All methods for inoculating pathogens mentioned in the text are based on the methods described in reference [36].

### 4.3. Genome-Wide Identification of the CsAGP Genes and Phylogenetic Tree Construction

Cucumber CsAGP protein sequences were retrieved from the ‘9930’ reference genome database (http://60.30.67.245:8070/#/download, accessed on 11 April 2025). AGPase family members were identified using two complementary strategies. First, the conserved NTP transferase domain (PF00483, https://pfam.xfam.org/, accessed on 11 April 2025) was used to search predicted *CsAGP* proteins with the Hidden Markov Model approach (version 3.4) (http://hmmer.org, accessed on 11 April 2025). Second, BLAST v2.441 searches were performed using AGP protein sequences from *A. thaliana*, *O. sativa*, *M. acuminata*, *Solanaceae* and *Maize as queries*. The AGP amino acid sequences were obtained from the TAIR database for *A. thaliana* (http://www.arabidopsis.org, accessed on 11 April 2025), the RGAP database for *O. sativa* (http://rice.plantbiology.msu.edu, accessed on 11 April 2025), the DH-Pahang genome database for *M. acuminata* (https://banana-genome-hub.southgreen.fr/downloads, accessed on 11 April 2025), the Solanaceae genomics database for *Solanaceae* (https://solgenomics.net/, accessed on 11 April 2025), and the Maize genomics database for *Maize* (https://maizegdb.org/, accessed on 11 April 2025). A phylogenetic tree for AGPs was constructed with 1000 bootstrap tests based on unrooted neighbor-joining using MEGA X software (version 10.2) [37].

### 4.4. Protein Property and Gene Structure Analysis

Theoretical molecular weight (MW) and isoelectric point (pI) values of AGP proteins were calculated using ExPAsy (version 3.0) [38]. Subcellular localization was predicted with the UniProt database (https://www.uniprot.org/, accessed on 12 April 2025) and Plant-mPLoc in Cell-PLoc (version 2.0) (http://www.csbio.sjtu.edu.cn/bioinf/Cell-PLoc-2/, accessed on 12 April 2025). Exon-intron structures were determined from genomic sequences using the Gene Structure Display Server (version 2.0) (http://gsds.gao-lab.org/, accessed on 12 April 2025) [39] and visualized with TBtools-II (version 2.371) [40]. Conserved protein motifs were identified with MEME (version 5.5.9) (http://meme-suite.org, accessed on 12 April 2025). A phylogenetic tree based on unrooted neighbor-joining was constructed with 1000 bootstrap tests using MEGA X software, and *CsAGP genes* were classified according to the topology of the phylogenetic trees [37].

### 4.5. Chromosomal Localization and Gene Duplication in CsAGP Genes

Chromosomal positions of *AGP* genes were visualized with TBtools based on genome annotation files [40]. MCscanX was used to perform tandem duplication analysis of AGPs in *Cucumis sativus*, *Arabidopsis thaliana*, *O. sativa*, *M. acuminata*, *Solanaceae* and *Maize*. Ka/Ks ratios were calculated with KaKs_calculator (version 2.0) using the YN model [41].

### 4.6. Prediction of Cis-Acting Elements in the CsAGP Gene Family

For promoter analysis, the 2000 bp upstream sequences of all five *CsAGP* genes were obtained from the cucumber genome database. Conserved cis-acting regulatory elements present in these regions were predicted with PlantCARE (http://bioinformatics.psb.ugent.be/webtools/plantcare/html/, accessed on 13 April 2025) [42].

### 4.7. Expression Profiling of CsAGP Genes

To investigate *CsAGP* transcriptional patterns, we retrieved relevant expression datasets from the cucumber genome database (http://60.30.67.245:8070/#/home, accessed on 20 April 2025) [36]. Furthermore, we used TBtools to analyze the expression patterns of *CsAGP* genes in spatial-temporal development, stress resistance, and disease resistance.

### 4.8. Expression Analysis of CsAGP Genes by qRT-PCR

To investigate whether the response of *CsAGP* genes to different pathogens is specific, we conducted expression analysis at various time points after inoculation with the three pathogens. For each time point, three biological replicates were harvested, with three leaves per replicate. Collected leaves were immediately frozen in liquid nitrogen and stored at −80 °C until use. Gene-specific primers were designed with Primer 3.0 according to *CsAGP* sequences (Table S1). Total RNA was extracted with the Plant RNA Extraction Kit (TaKaRa MiniBEST, Beijing, China), and first-strand cDNA was synthesized using HiScript III RT SuperMix for qPCR (Vazyme Biotech, Beijing, China). Real-time PCR was performed with ChamQ Universal SYBR qPCR Master Mix (Vazyme Biotech, Beijing, China) under the following program: 95 °C for 3 s, followed by 39 cycles of 95 °C for 10 s, 60 °C for 30 s, and 72 °C for 20 s. Each treatment was performed with three biological and technical replicates. *Actin1* (*CsaV4_3G003833*) was used as a reference gene, and the 2^−ΔΔCt^ algorithm was used to calculate gene expression levels. Statistical analyses were performed with GraphPad Prism (version 9.0) and Microsoft Excel 2021.

### 4.9. AGPase Enzyme Activity and Transmission Electron Microscopy (TEM)

When the first true leaf of seedlings was fully expanded, three biological replicates were taken separately, with three plants per replicate. A total of 0.1 g of leaves was collected and added to 1 mL of extraction solution, then ground into a homogenate in an ice bath. The measurement was then carried out according to the method described in kit BC0430 (Solarbio, Beijing, China).

When the first true leaf of seedlings was fully expanded, fresh tissue samples were immediately placed in electron microscopy fixative and cut into 1 mm^3^ pieces. After vacuum pumping to submerge the samples, they were fixed at room temperature for 2 h and stored protected from light at 4 °C. Following fixation, the samples were rinsed with 0.1 M phosphate buffer (pH 7.4), fixed with 1% osmium tetroxide protected from light for 7 h, and rinsed again with buffer. Subsequently, the samples were dehydrated stepwise using gradient ethanol (30% to 100%) and acetone, followed by gradient infiltration and embedding with 812 epoxy resin, and polymerized at 60 °C for 48 h. Ultrathin sections of 60–80 nm were cut using an ultramicrotome, collected on 150-mesh copper grids, and double-stained with uranyl acetate and lead citrate. The sections were then observed and imaged under a transmission electron microscope [43].

### 4.10. Plasmid Construction and Plant Transformation

For CRISPR/Cas9-mediated cucumber transformation of *CsAGP4*, sgRNA sequences targeting *CsAGP4* were computationally designed through the CRISPR-GE platform [44]. Complementary oligonucleotides corresponding to selected guide sequences were annealed and directionally cloned into the BsaI-digested pKSE402 binary vector using T4 DNA ligase (New England Biolabs, Beijing, China). The recombinant plasmid was subsequently electroporated into *Agrobacterium tumefaciens* strain EHA105 for cucumber transformation. Primary transformants were screened through PCR amplification of the target region followed by bidirectional Sanger sequencing to verify editing events. Confirmed heterozygous mutants were advanced through self-fertilization cycles to isolate T2-generation homozygous lines, which were subsequently subjected to comprehensive phenotypic and molecular analyses. Positive clones were verified by colony PCR and Sanger sequencing (Sangon Biotech, Beijing, China). Primer sequences are provided in Supplementary Table S1.

## 5. Conclusions

In this study, we systematically characterized the *AGPase* gene family and identified *CsAGP4* as a WMV-responsive isoform whose expression is preferentially activated in the resistant genotype. Hormone assays showed that ABA and JA rapidly remodel the transcriptional landscape of *CsAGP* genes during WMV infection and synergistically enhance *CsAGP4* induction, indicating that *CsAGP4* is not merely a constitutive metabolic enzyme but is integrated into classical antiviral hormone-signaling pathways. CRISPR/Cas9-generated knockout mutants demonstrated that *CsAGP4* is indispensable for starch granule formation, ADP-glucose pyrophosphorylase activity and effective restriction of WMV accumulation at the seedling stage. Loss of *CsAGP4* not only blocked starch biosynthesis but also markedly increased disease severity, showing that AGPase-mediated carbon allocation is an important component of antiviral immunity.

## Figures and Tables

**Table 1:** Characteristics of *CsAGP* gene members in cucumber.

Proposed Gene Name	Gene ID	Subcellular Localization of Predicted Protein	Isoelectric Point (pI) of Predicted Protein	Molecular Weight (Da) of Predicted Protein	Number of Amino Acids of Predicted Protein
*CsAGP1*	*CsaV4_2G001288*	Chloroplast	8.21	58,630.29	527
*CsAGP2*	*CsaV4_3G001409*	Chloroplast	8.8	57,640.16	523
*CsAGP3*	*CsaV4_3G004518*	Chloroplast	5.86	59,208.46	532
*CsAGP4*	*CsaV4_6G001323*	Chloroplast	9.09	59,906.89	536
*CsAGP5*	*CsaV4_7G000260*	Chloroplast	7.59	56,917.85	521

## Data Availability

All relevant data are within this article and its Supplementary Materials. Further inquiries can be directed to the first authors.

## Supplementary Materials

The following supporting information can be downloaded at: https://www.mdpi.com/article/10.3390/ijms27114764/s1.
