# Supplementary material for: Genome-Wide Analysis of AGPase Identifies CsAGP4 as a Regulator of Watermelon Mosaic Virus Resistance in Cucumber
Source: Int J Mol Sci. 2026 May 25;27(11):4764. doi: 10.3390/ijms27114764 (PMC13256830; doi:10.3390/ijms27114764)
Supplement: Supplementary file 1 [file ijms-27-04764-s001.zip › Supplementary Table.pdf]

Supplementary Table S1. The primers used in this article.

classification	Primer name	Primer sequence
qRT-PCR	QpcrCsAGP1-F	GCCTAATGTTTCAAAGCAGCC
	QpcrCsAGP1-R	GCGGGTGTTGCTGATCTTTT
	QpcrCsAGP2-F	TGTTTGCATGTCTCTCACGA
	QpcrCsAGP2-R	GAATGATCGCCACCACAGTC
	QpcrCsAGP3-F	CCCGGTGTTGCTTATTCTGT
	QpcrCsAGP3-R	AGCACCTCCACCCAGTATAA
	QpcrCsAGP4-F	TATACTTGGAGGAGGAGCGG
	QpcrCsAGP4-R	TCGGCACATCAATCAGTCTG
	QpcrCsAGP5-F	TTCTGGTTTGGGATCACGAC
	QpcrCsAGP5-R	GCCCAGAATAATCCCAAGCA
CRISPR	CRISPR-F	ATTGCTCCAACATGCTACAGGTG
	CRISPR-R	AAACCACCTGTAGCATGTTGGAG
	JC-F	CACTCAACACATACAACCA
	JC-R	GAAAGGAGACTTGCATTATTGC
