# Supplementary material for: Genome-Wide Analysis of AGPase Identifies CsAGP4 as a Regulator of Watermelon Mosaic Virus Resistance in Cucumber
Source: Int J Mol Sci. 2026 May 25;27(11):4764. doi: 10.3390/ijms27114764 (PMC13256830; doi:10.3390/ijms27114764)
Supplement: Supplementary file 1 [file ijms-27-04764-s001.zip › Figure S1.pdf]

```
          1    10   20   30   40   50   60   70   80   90   100  110  120  130
9930      ATGGTAGCCTCTGCCGCTGCCCACTTGCACACTGCAATGGCATTGCATAAAGTCACCAACTCCAACATGCTACAGGTGAGGTTCTGTAATGGAGAATTCATGGGGAAGAAGCTCACATTTCAACAGAGGA
Poinsett  ATGGTAGCCTCTGCCGCTGCCCACTTGCACACTGCAATGGCATTGCATAAAGTCACCAACTCCAACATGCTACAGGTGAGGTTCTGTAATGGAGAATTCATGGGGAAGAAGCTCACATTTCAACAGAGGA
Consensus ATGGTAGCCTCTGCCGCTGCCCACTTGCACACTGCAATGGCATTGCATAAAGTCACCAACTCCAACATGCTACAGGTGAGGTTCTGTAATGGAGAATTCATGGGGAAGAAGCTCACATTTCAACAGAGGA

          131  140  150  160  170  180  190  200  210  220  230  240  250  260
9930      AACAAAATATTAATTACATTCCAAATCTCAAACGGAAACTTTGTATCTCTTCTCTTATTGCCGATATAGCAGCTGAATCCAAGTTGAGAGGATTGAACATGGAAAAACGTGACTCGAGAACGGTGGTGGC
Poinsett  AACAAAATATTAATTACATTCCAAATCTCAAACGGAAACTTTGTATCTCTTCTCTTATTGCCGATATAGCAGCTGAATCCAAGTTGAGAGGATTGAACATGGAAAAACGTGACTCGAGAACGGTGGTGGC
Consensus AACAAAATATTAATTACATTCCAAATCTCAAACGGAAACTTTGTATCTCTTCTCTTATTGCCGATATAGCAGCTGAATCCAAGTTGAGAGGATTGAACATGGAAAAACGTGACTCGAGAACGGTGGTGGC

          261  270  280  290  300  310  320  330  340  350  360  370  380  390
9930      GGTTATACTTGGAGGAGGAGCGGGTACTCGTTTGTTCCCGCTCACTAAACAGAGAGCAAAACCGGCGGTTCCGATCGGTGGGGCTTACAGACTGATTGATGTGCCGATGAGCAATTGCATAAACAGTGGA
Poinsett  GGTTATACTTGGAGGAGGAGCGGGTACTCGTTTGTTCCCGCTCACTAAACAGAGAGCAAAACCGGCGGTTCCGATCGGTGGGGCTTACAGACTGATTGATGTGCCGATGAGCAATTGCATAAACAGTGGA
Consensus GGTTATACTTGGAGGAGGAGCGGGTACTCGTTTGTTCCCGCTCACTAAACAGAGAGCAAAACCGGCGGTTCCGATCGGTGGGGCTTACAGACTGATTGATGTGCCGATGAGCAATTGCATAAACAGTGGA

          391  400  410  420  430  440  450  460  470  480  490  500  510  520
9930      ATCAACAAGGTTTACATTCTCACACAGTTCAATTCCGCATCGCTCAATAGGCATCTCGCTCGCGCTTACAATTTCGGTAGCGGAGTCACCTTAGGAGATGGTTTCGTTGAGGTTCTAGCAGCTACTCAAA
Poinsett  ATCAACAAGGTTTACATTCTCACACAGTTCAATTCCGCATCGCTCAATAGGCATCTCGCTCGCGCTTACAATTTCGGTAGCGGAGTCACCTTAGGAGATGGTTTCGTTGAGGTTCTAGCAGCTACTCAAA
Consensus ATCAACAAGGTTTACATTCTCACACAGTTCAATTCCGCATCGCTCAATAGGCATCTCGCTCGCGCTTACAATTTCGGTAGCGGAGTCACCTTAGGAGATGGTTTCGTTGAGGTTCTAGCAGCTACTCAAA

          521  530  540  550  560  570  580  590  600  610  620  630  640  650
9930      CTCCAGGAGAGGCCGGGAAGCGGTGGTTTCAGGGGACTGCCGATGCCGTACGACAGTTCCATTGGCTTTTTGAGGATGCAAGAAGCAAGGACATTGAAGATGTGTTGATTCTTTCTGGGGATCACTTGTA
Poinsett  CTCCAGGAGAGGCCGGGAAGCGGTGGTTTCAGGGGACTGCCGATGCCGTACGACAGTTCCATTGGCTTTTTGAGGATGCAAGAAGCAAGGACATTGAAGATGTGTTGATTCTTTCTGGGGATCACTTGTA
Consensus CTCCAGGAGAGGCCGGGAAGCGGTGGTTTCAGGGGACTGCCGATGCCGTACGACAGTTCCATTGGCTTTTTGAGGATGCAAGAAGCAAGGACATTGAAGATGTGTTGATTCTTTCTGGGGATCACTTGTA

          651  660  670  680  690  700  710  720  730  740  750  760  770  780
9930      TCGAATGGACTACATGGACTTTGTTCAAAATCACCGGCAGAGTGGTGCAGACATCACTCTTTCTTGTATCCCAATAGATGACAGTCGAGCGTCGGATTTTGGGCTAATGAAGATCGACAATAGCGGAAGG
Poinsett  TCGAATGGACTACATGGACTTTGTTCAAAATCACCGGCAGAGTGGTGCAGACATCACTCTTTCTTGTATCCCAATAGATGACAGTCGAGCGTCGGATTTTGGGCTAATGAAGATCGACAATAGCGGAAGG
Consensus TCGAATGGACTACATGGACTTTGTTCAAAATCACCGGCAGAGTGGTGCAGACATCACTCTTTCTTGTATCCCAATAGATGACAGTCGAGCGTCGGATTTTGGGCTAATGAAGATCGACAATAGCGGAAGG

          781  790  800  810  820  830  840  850  860  870  880  890  900  910
9930      GTTATTTCATTCAGTGAAAAGCCTAGAGGGAAAGACCTGAAGGCGATGGAAGTGGACACAACAGTTTTAGGACTGTCAAAGGATGAGGCATTGAGAAAGCCGTACATAGCTTCCATGGGAGTTTATATCT
Poinsett  GTTATTTCATTCAGTGAAAAGCCTAGAGGGAAAGACCTGAAGGCGATGGAAGTGGACACAACAGTTTTAGGACTGTCAAAGGATGAGGCATTGAGAAAGCCGTACATAGCTTCCATGGGAGTTTATATCT
Consensus GTTATTTCATTCAGTGAAAAGCCTAGAGGGAAAGACCTGAAGGCGATGGAAGTGGACACAACAGTTTTAGGACTGTCAAAGGATGAGGCATTGAGAAAGCCGTACATAGCTTCCATGGGAGTTTATATCT

          911  920  930  940  950  960  970  980  990  1000 1010 1020 1030 1040
9930      TCAAAAAGGAGATACTTCTAAACATTTTGAGATGGCGCTTCCCAACTGCGAATGACTTTGGATCAGAGATAATCCCTTTCTCAGCAAGAGAATTCTTGATGAAGGCATATTTATTTAATGATTATTGGGA
Poinsett  TCAAAAAGGAGATACTTCTAAACATTTTGAGATGGCGCTTCCCAACTGCGAATGACTTTGGATCAGAGATAATCCCTTTCTCAGCAAGAGAATTCTTGATGAAGGCATATTTATTTAATGATTATTGGGA
Consensus TCAAAAAGGAGATACTTCTAAACATTTTGAGATGGCGCTTCCCAACTGCGAATGACTTTGGATCAGAGATAATCCCTTTCTCAGCAAGAGAATTCTTGATGAAGGCATATTTATTTAATGATTATTGGGA

          1041 1050 1060 1070 1080 1090 1100 1110 1120 1130 1140 1150 1160 1170
9930      AGACATAGGGACTATTAGATCCTTCTTTGAGGCAAATCTTGCGCTGACAGAGCAGCCACCAAGATTTAGCTTCTATGACGAAACAAAACCAATCTACACTTCAAGAAGAAATCTACCACCAACGAAGATT
Poinsett  AGACATAGGGACTATTAGATCCTTCTTTGAGGCAAATCTTGCGCTGACAGAGCAGCCACCAAGATTTAGCTTCTATGACGAAACAAAACCAATCTACACTTCAAGAAGAAATCTACCACCAACGAAGATT
Consensus AGACATAGGGACTATTAGATCCTTCTTTGAGGCAAATCTTGCGCTGACAGAGCAGCCACCAAGATTTAGCTTCTATGACGAAACAAAACCAATCTACACTTCAAGAAGAAATCTACCACCAACGAAGATT

          1171 1180 1190 1200 1210 1220 1230 1240 1250 1260 1270 1280 1290 1300
9930      GACAATTGCAAGATTGTTGATTCAATCATATCTCATGGATGTTTCTTGACTAACAGTTTCATAGATCACAGTGTCGTTGGTATTCGATCTCGCATAAACTCTAACGTTCACCTCAAGGACACAGTGATGC
Poinsett  GACAATTGCAAGATTGTTGATTCAATCATATCTCATGGATGTTTCTTGACTAACAGTTTCATAGATCACAGTGTCGTTGGTATTCGATCTCGCATAAACTCTAACGTTCACCTCAAGGACACAGTGATGC
Consensus GACAATTGCAAGATTGTTGATTCAATCATATCTCATGGATGTTTCTTGACTAACAGTTTCATAGATCACAGTGTCGTTGGTATTCGATCTCGCATAAACTCTAACGTTCACCTCAAGGACACAGTGATGC

          1301 1310 1320 1330 1340 1350 1360 1370 1380 1390 1400 1410 1420 1430
9930      TTGGAGCTGACTTCTATGAAACTGAAGGGGAAGTCGCTGCACTTCTAGCTGAGGGAAGAGTGCCAATTGGAATTGGAGAAAATACAAAAATCAAGGACTGCATCATTGACAAAAATGCCAGAATAGGGAA
Poinsett  TTGGAGCTGACTTCTATGAAACTGAAGGGGAAGTCGCTGCACTTCTAGCTGAGGGAAGAGTGCCAATTGGAATTGGAGAAAATACAAAAATCAAGGACTGCATCATTGACAAAAATGCCAGAATAGGGAA
Consensus TTGGAGCTGACTTCTATGAAACTGAAGGGGAAGTCGCTGCACTTCTAGCTGAGGGAAGAGTGCCAATTGGAATTGGAGAAAATACAAAAATCAAGGACTGCATCATTGACAAAAATGCCAGAATAGGGAA

          1431 1440 1450 1460 1470 1480 1490 1500 1510 1520 1530 1540 1550 1557
9930      AAATGTTGTTCTTGCTAATTCAGAGGGGGTTCAAGAGGCAGATAGGTCTTCAGAAGGGTTTTACATCCGCTCAGGAATTACCATTATACTAAGGAACTCAGTTATTAAAGATGGGTTTGTGATTTAG
Poinsett  AAATGTTGTTCTTGCTAATTCAGAGGGGGTTCAAGAGGCAGATAGGTCTTCAGAAGGGTTTTACATCCGCTCAGGAATTACCATTATACTAAGGAACTCAGTTATTAAAGATGGGTTTGTGATTTAG
Consensus AAATGTTGTTCTTGCTAATTCAGAGGGGGTTCAAGAGGCAGATAGGTCTTCAGAAGGGTTTTACATCCGCTCAGGAATTACCATTATACTAAGGAACTCAGTTATTAAAGATGGGTTTGTGATTTAG
```
